# The anticancer effects of ferulic acid is associated with induction of cell cycle arrest and autophagy in cervical cancer cells

**DOI:** 10.1186/s12935-018-0595-y

**Published:** 2018-07-13

**Authors:** Jinhua Gao, Hui Yu, Weikang Guo, Ying Kong, lina Gu, Qi Li, Shanshan Yang, Yunyan Zhang, Yaoxian Wang

**Affiliations:** 1 Department of Gynecology, Harbin Medical University Cancer Hospital, No. 150 Haping Road, Nangang District, Harbin, 150081 Heilongjiang China; 2 Department of Cardiopulmonary Function, Harbin Medical University Cancer Hospital, Harbin, 150081 Heilongjiang China; 3 Department of Internal Medicine, The Second Affiliated Hospital of Heilongjiang University of Chinese Medicine, Harbin, 150001 Heilongjiang China

**Keywords:** Ferulic acid, Cervical cancer, Cell cycle, Autophagy, Invasion

## Abstract

**Background:**

Ferulic acid (4-hydroxy-3-methoxycinnamic acid, FA) is a hydroxycinnamic acid derived from a rich polyphenolic compound. This study aimed to investigate the effect of ferulic acid (4-hydroxy-3-methoxycinnamic acid; FA) on cell proliferation, invasion, apoptosis, and autophagy in Hela and Caski cervical carcinoma cell lines.

**Methods:**

The cell proliferation of FA in Hela and Caski cells were detected by MTT assay. The cell invasion of FA in Hela and Caski cells were detected by Transwell assay. Subsequently, MMP-9 mRNA expression for cell invasion was detected by RT-PCR. Additionally, cell cycle and apoptosis were assayed using flow cytometry. Expression levels of 7 proteins for both cell cycle and autophagy were measured by Western blot analysis.

**Results:**

After treated with FA (2.0 mM) for 48 h, the inhibition rates of FA in Hela and Caski cells were 88.3 and 85.4%, respectively. In addition, FA inhibited cell invasion through reducing MMP-9 mRNA expression. FA induced arrest in G0/G1 phase of the cell cycle in Hela and Caski cells with dose dependent (P < 0.05). Meanwhile, FA induced the cell cycle-related proteins expression such as p53 and p21, and reduced Cyclin D1 and Cyclin E levels. Moreover, FA decreased the autophagy-related proteins such as LC3-II, Beclin1 and Atg12-Atg5 in a dose-dependent manner.

**Conclusion:**

FA can significantly inhibit cell proliferation and invasion in Hela and Caski cells. It might be acted as an anti-cancer drug through inhibiting the autophagy and inducing cell cycle arrest in human cervical carcinoma cells.

## Background

Cervical cancer is the fourth common cause of death in women worldwide [1]. Nearly 530,000 women with cervical cancer were diagnosed and 26,6000 women died from cervical cancer worldwide in 2012 [2]. Generally, human papillomavirus infection (HPV) causes more than 90% of cases [3]. However, most people with HPV infection do not develop cervical cancer. HPV 16 and 18 are the main cause for cervical cancer globally, while HPV 31 and 45 are the second causes for another patient [4]. At present, pelvic surgery is the main treatment for early cervical cancer in the world [5]. Additionally, chemotherapy can be used to treat cervical cancer. Chemotherapy has become a common method in the adjuvant therapy of women with early cervical cancer, especially those patients with advanced or recurrent cancer [6]. However, a wide variety of chemotherapy drugs for the treatment of cervical cancer have many side effects such as neurotoxicity, which lead to the limitations of its application and function [7]. Therefore, it is a primary concern to develop a novel drug with minimal side effects for preventing and treating the cervical cancer.

Ferulic acid (4-hydroxy-3-methoxycinnamic acid, FA) is a hydroxycinnamic acid and an abundant phenolic phytochemical in vegetables and fruits, which has antioxidant and antitumor activities [8]. FA has been identified in Chinese medicine herbs such as *Angelica sinensis*, *Cimicifuga heracleifolia* and *Ligusticum chuangxiong* [9, 10]. In the previous studies, FA is an effective antioxidant agent that protects DNA from oxidative damage and prevents lipid peroxidation through reducing oxidative stress [11]. In many tumor cell lines such as human osteosarcoma, human glioblastoma (U87MG), and prostate cancer, FA can induce cytotoxicity [12–14]. Due to the inhibition of cyclooxygenase-2, FA is considered to be an anti-proliferative agent [15]. In addition, FA has radioprotective function on human lymphocytes in previous studies, and FA may induce cell apoptosis in cancer [16]. Besides, studies also found that FA inhibits the cell activities and enhanced oxidative DNA damage in HeLa and ME-180 human cervical cancer cells [17]. However, the current research on the inhibitory effect and mechanism of FA in human cervical cancer cells is unclear.

Therefore, this study aimed to explore the effect of FA on Hela and Caski human cervical cancer cells as well as its molecular mechanism. In thist study, we study the changes of FA on genes and proteins expression, cell proliferation, invasion, cycle and apoptosis in Hela and Caski human cervical cancer cell.

## Materials and methods

### Chemicals

FA was purchased from Meilunbio (Dalian Meilun Biotechnology Co., LTD. Liaoning, China). Antibodies for P53, P21, Cyclin D1, Cyclin E, Beclin-1, LC3-II, Atg12-Atg5 and β-actin used for Western blot analysis were purchased from Wanleibio (Shenyang, Liaoning, China). Super moloney-murine leukemia virus (M-MLV) reverse transcriptase for fluorescence quantification was purchased from BioTeke (Beijing, China) and RNA simple Total RNA Kit was purchased from TIANGEN (Beijing, China).

### Cell culture

Hela and Caski cells were purchased from Shanghai Cell Bank of Chinese Academy of Sciences. Hela cells were incubated in DMEM medium with 40% fetal bovine serum (FBS), and Caski cells were incubated in RPMI-1640 medium containing 10% FBS. These cells were seed in 96-well plate and placed in an incubator at 37 °C and 5% CO_2_.

### Cell proliferation assay

MTT assay was used to assay the cell proliferation using various concentrations of FA (0.5, 1.0, 1.5, 2.0 mM). The cells who were treated without FA were the control group. Each experiment was performed in triplicate. After cultured for 48 h, MTT at a concentration of 0.2 mg/ml was added to the plates for 4 to 6 h. Then, cell viability was measured using an MTT mixture according to manufacturer’s instruction. Formazan formation was quantified spectrophotometrically at 490 nm (reference wavelength 630 nm) using a microplate reader. As follows: viability % = (OD value of experimental group/OD value of control group) × 100%.

### Real-time PCR

Total RNA was extracted from the control and FA-treated cells using the Total RNA Extraction Kit following the manufacturer’s instructions. cDNA was synthesized using 1 µL M-MLV reverse transcriptase. Subsequently, Atg5, Beclin-1, and MMP-9 expression levels were detected with real-time PCR quantification based on SYBR Green PCR Master Mix (Solarbio, Beijing, China), and melting curves were acquired after amplification. β-actin was set as a reference gene. The primer sequence is shown in Table 1.

**Table 1:** Primer sequences of the genes used in this study

Genes	Primer sequences
MMP-9	F: 5′-AGTCCACCCTTGTGCTCTTC-3′
	R: 5′-GCCACCCGAGTGTAACCAT-3′
Atg5	F: 5′-GTATCATCCCACAGCCAAC-3′
	R: 5′-GCAAAGTAAGACCAGCCC-3′
Beclin-1	F: 5′-AACCAGATGCGTTATGCC-3′
	R: 5′-CGTAAGGAACAAGTCGGTAT-3′
β-actin	F: 5′-CTTAGTTGCGTTACACCCTTTCTTG-3′
	R: 5′-CTGTCACCTTCACCGTTCCAGTTT-3′

### Western blotting

Protein expression levels of P53, P21, Cyclin D1, Cyclin E, Beclin-1, LC3-II, and Atg12–Atg5 were determined by Western blotting. β-actin is a reference protein. The protocol was performed according to the previous study [18]. The primary antibodies (1:1000 dilution) were purchased from Meilunbio, then the sheep anti-rabbit secondary antibodies (1:5000) were used. The OD values of bands were visualized using Gel-Pro-Analyzer software.

### Transwell invasion assay

The Transwell compartments (Corning, USA) were placed into a 24-well plate and pre-coated with 50 μL diluted Matrigel. At first, Hela or Caski cells were cultured in the upper chamber including DMEM or RPMI-1640 medium with free FBS, followed by treatment with various concentrations of FA (0, 1.0, 2.0, 4.0 mM). Subsequently, DMEM or RPMI-1640 medium supplemented with FBS was added to the lower chamber, and cells were performed to migrate for 48 h at 37 °C. The cells in the lower chamber were stained with crystal violet and then were counted under a microscope.

### Cell cycle and apoptosis

The Hela and Caski cells were treated with various concentrations of FA (0, 1.0, 2.0, 4.0 mM). After 48 h, cells were collected and washed twice with cold PBS. Then cells were incubated in a 1 mL of mixed solution including 20 mg/mL of propidium iodide (PI) and 10 U/mL of RNaseA (KGA214, KeyGen, Nanjing, China) for 30 min at room temperature. Cell cycle was assayed through the ModFit software after fluorescence-activated cell sorting (FACS). For apoptosis analysis, the Annexin V-FITC/PI apoptosis detection kit (KeyGEN Bio TECH, Nanjing, China) was used following the manufacturer’s instruction.

### Statistical analysis

All data were presented as mean ± standard deviation. The differences between two groups were detected using the two sample independent *T* test. The one-way ANOVA was applied for comparison among three or more groups following LSD method. The linear regression method was used to evaluate the dose–effect relationship (R^2^). For all the analysis, P < 0.05 was considered significant difference. SPSS 19.0 (SPSS Inc., NY, USA) was used in the present study.

## Results

### Anti-proliferation activity of FA on Hela and Caski cervical cancer cells

**Fig. 1:**
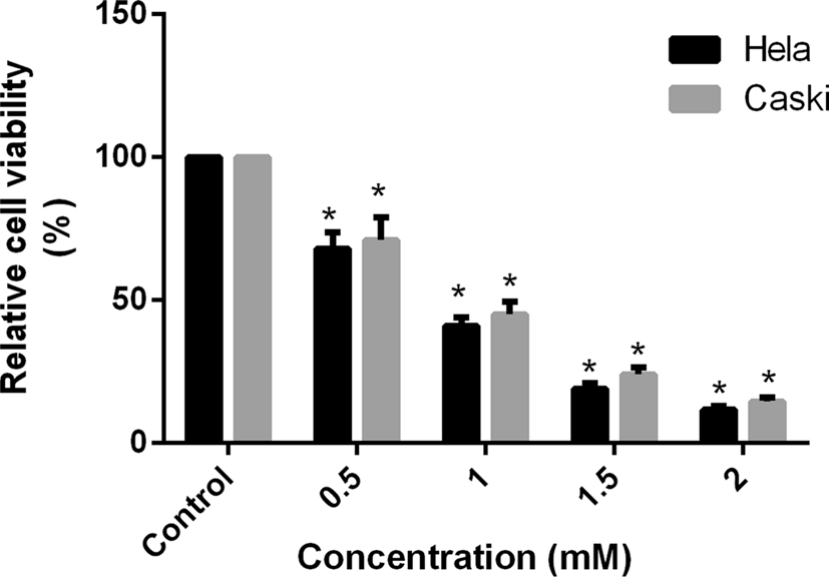
Effect of the different concentrations of FA on cell proliferation activity in Hela and Caski cells. *P < 0.05 indicates that cell proliferation activity was significantly decreased when compared with the control group

Cell viability of Hela and Caski cells were significantly decreased along with the increasing concentration. The proliferation rate of FA with different concentration in Hela cells were 67.97, 41.07, 19.23, and 11.67% respectively, and that in Caski cells were 70.97, 45.03, 24.03, and 14.63% when compared with the control group (Fig. 1). These results indicated that FA inhibited cell proliferation in Hela and Caski cells through a concentration-dependent manner (${\text{R}}^{ 2}_{\text{Hela}} = \, 0. 9 5,{\text{ P }} < 0.0 1;{\text{ R}}^{ 2}_{\text{Caski}} = \, 0. 9 6,{\text{ P }} < 0.0 1$). At 2.0 mM, the cell viabilities in FA groups for 48 h were significantly reduced and were up to 88.3 and 85.4%, respectively (Fig. 1).

### FA inhibited cell invasion

**Fig. 2:**
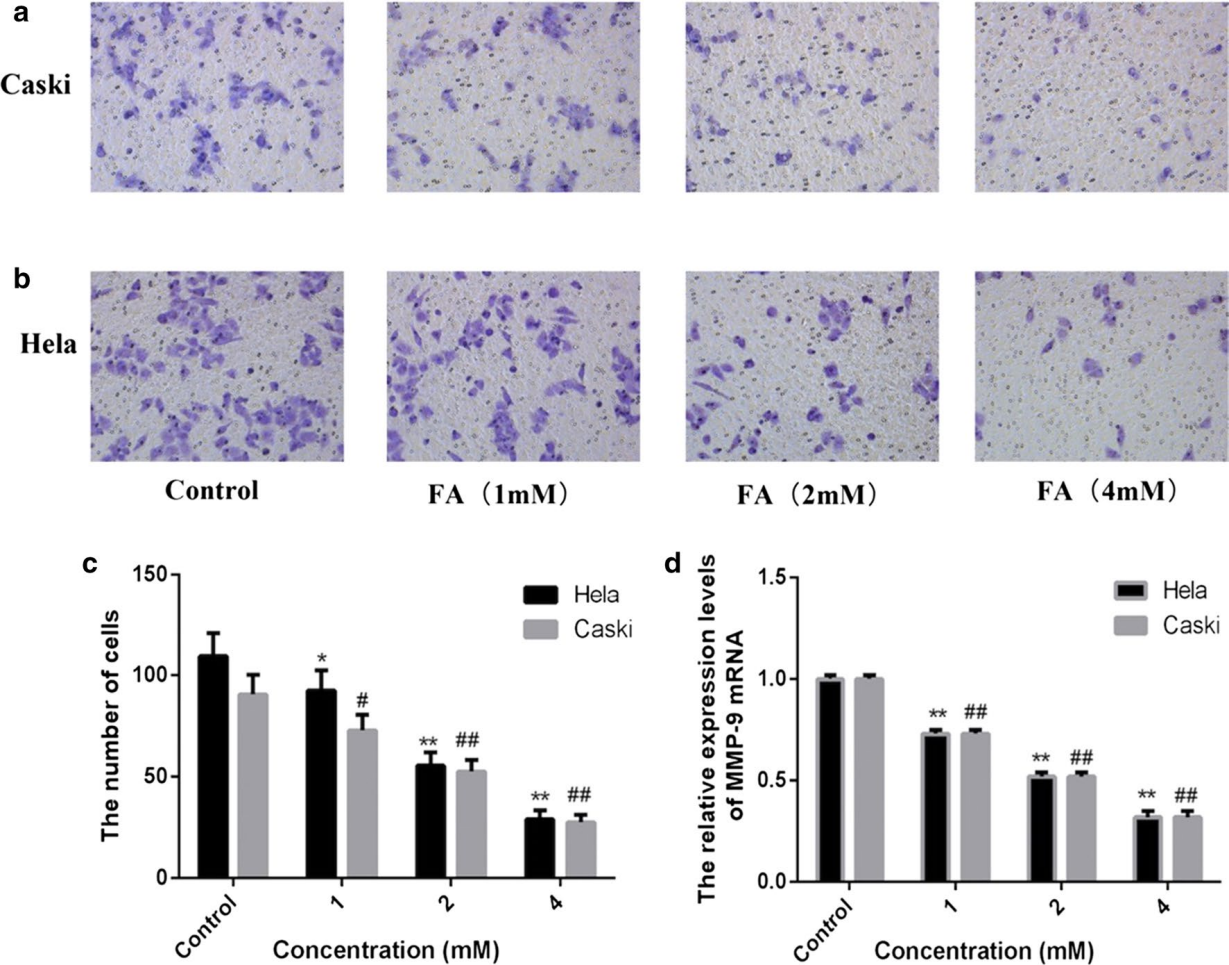
FA induced morphological chang in Caski cells (**a**). The morphology of the Hela cells was examined using a phase contrast microscope after treatment with FA (**b**). Effect of FA on cell invasion in Hela and Caski cells. **c** MMP-9 mRNA expression in Hela and Caski cells; d the effect of FA (1.0, 2.0 and 4.0 mM) on cell invasion in Hela and Caski cells. *P < 0.05 indicates that there are significantly different compared with the control group; **P < 0.01

Hela cells and Caski cells were incubated with FA (0,1.0,2.0,4.0 mM). The morphology of the cells was examined using a phase contrast microscope. In the presence of FA, Caski cells and Hela cells showed a circular morphology, with a small amount of contraction and nuclear condensation, and a portion of cells showed swelling, cell membrane lysis and organelle breakdown, indicating the cytotoxicity of Caski cells and Hela cells induced by FA (Fig. 2a, b). Subsequently, Transwell chambers were used to detect the effect of FA on cell invasion in those cells. The mean number of cells across to the basement membrane of Transwell chamber was decreased while the concentration of FA was reduced after Hela cells treatment with FA for 48 h (R2 = 0.93, P < 0.01, Fig. 2c). Similarly, the mean number of cells was also decreased while the concentration of FA was reduced after Caski cell treatment with FA for 48 h (Fig. 2c). The mRNA level of MMP-9 was detected by fluorescence quantitative PCR. The expression levels of MMP-9 mRNA (FHela = 603.35, P < 0.01; FCaski = 1988.07, P < 0.01) were significantly reduced in 4.0 mM FA group for 48 h and have a dose-dependent relationship (R2Hela = 0.99, P < 0.01; R2Caski = 0.96, P < 0.01; Fig. 2d).

### FA induced arrest in G0/G1 phase

**Fig. 3:**
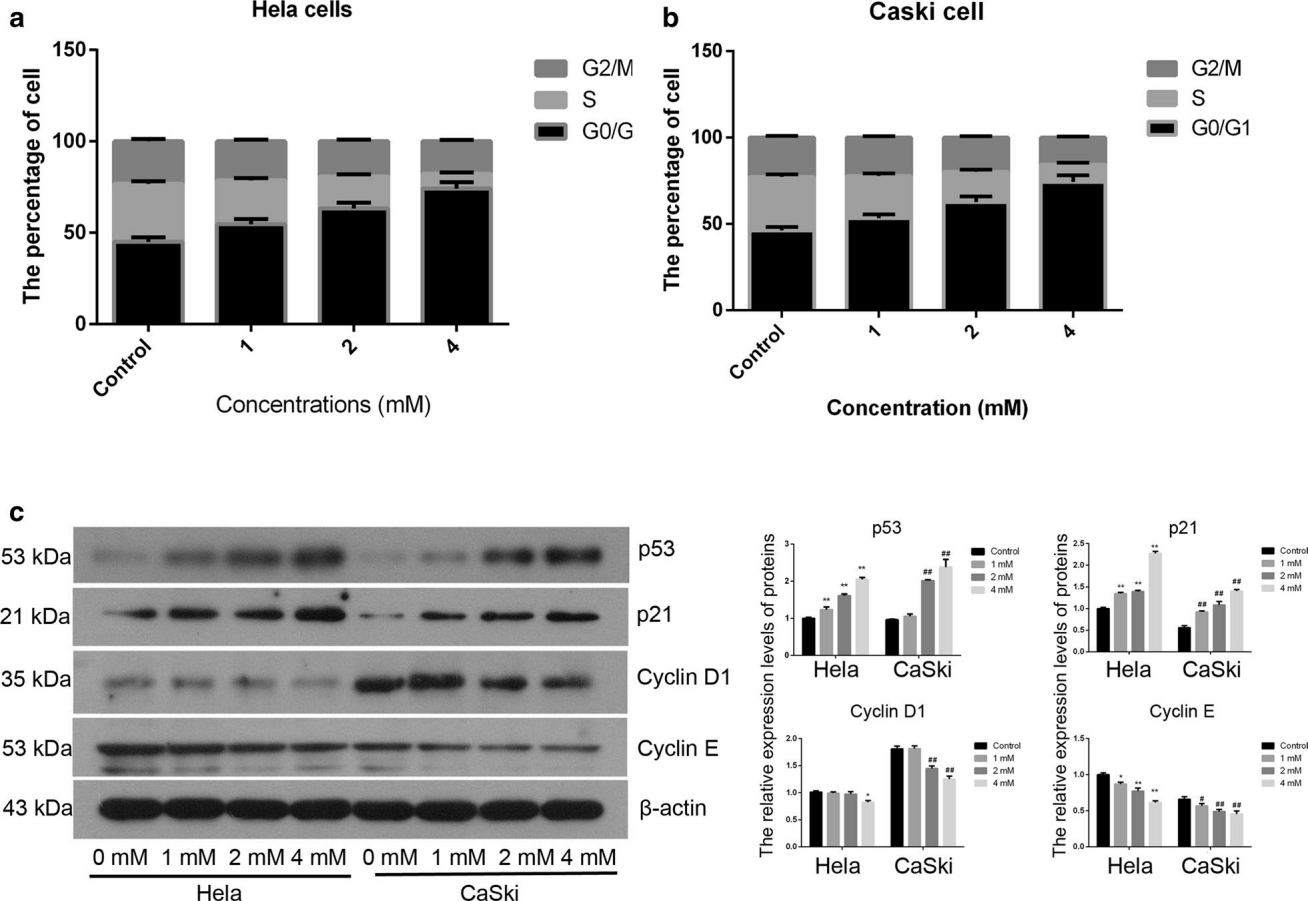
FA induced cell-cycle arrest in G0/G1 phase in Hela and Caski cells. **a** The rate of Hela cells in G0/G1 phase was increased in FA groups; **b** the rate of Caski cells in G0/G1 phase was increased in FA groups; **c** the expression levels of cell cycle related proteins such as P53, P21, Cyclin D1, Cyclin E were determined by Western blotting. β-actin is a reference protein. *P < 0.05 indicates that there are significantly different compared with the control group

In Hela cells, FA significantly induced G0/G1 phase arrest at 1.0, 2.0 and 4.0 mM, especially at the higher concentration (Fig. 3). Similar results were observed in Caski cells exposure to FA. At 4.0 mM of FA, the rate of Hela cells in G0/G1 phase was increased from 45.10 to 74.20% (F_Hela_ = 53.64, P < 0.01, Fig. 3a), and that in Caski cells was increased from 46.25 to 74.30% (F_Caski_ = 49.86, P < 0.01, Fig. 3b). In addition, FA induced Hela (R^2^ = 0.95, P < 0.01) and Caski (R^2^ = 0.94, P < 0.01) cell-cycle arrest in G0/G1 phase with a dose-dependent manner. Besides, P53 and P21 protein levels were increased in FA groups. When Hela cells were exposed to 4.0 mM of FA, P53 and P21 protein levels were 2.05 and 2.27 times as high as the control (P < 0.01, Fig. 3c). Similarly, P53 and P21 protein levels were 2.50 and 2.51 times as high as the control in Caski cells (P < 0.01, Fig. 3c). Moreover, CyclinD1 and CyclinE1 levels were reduced in the FA groups for 48 h (Fig. 3c).

#### The effect of FA on cell apoptosis

**Fig. 4:**
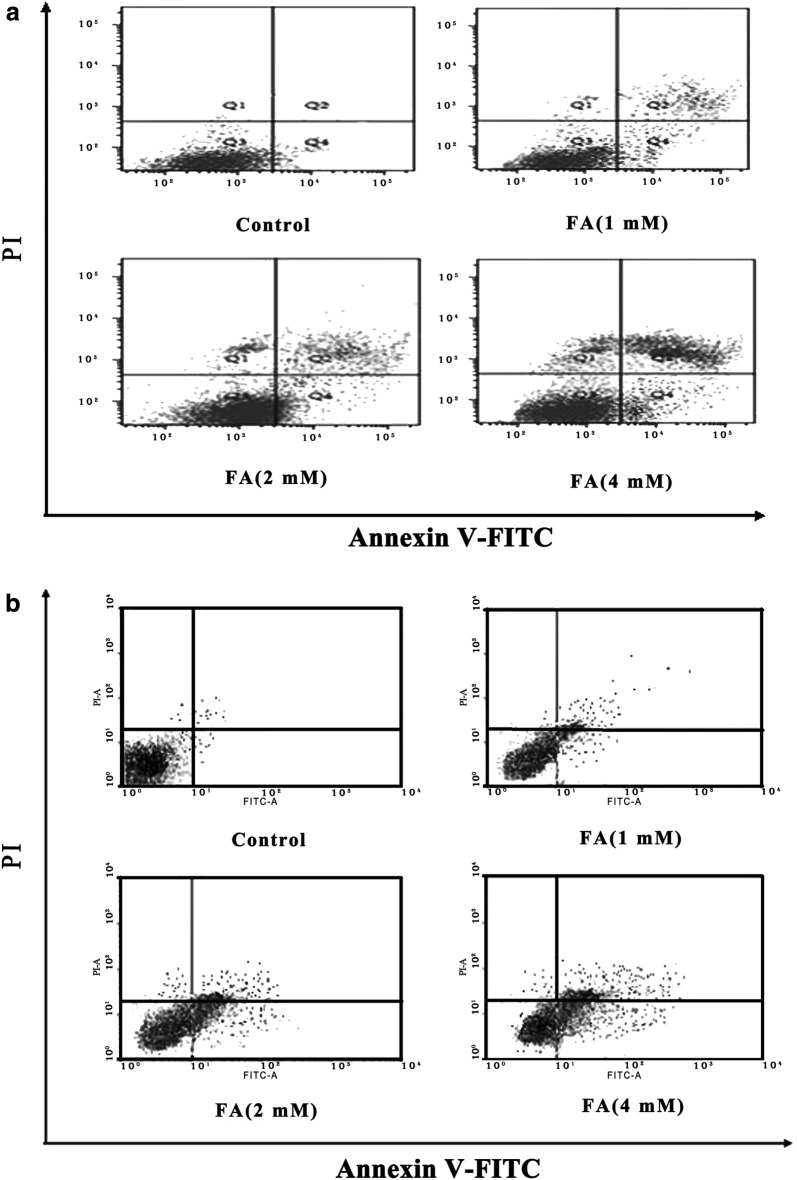
Effect of different concentrations of FA on cell apoptosis in Hela (**a**) and Caski (**b**) cells. *P < 0.05 indicates that there are significantly different compared to the control group

It was observed that cell apoptosis in the both of cells were induced after exposure to FA when compared with the control group. The apoptotic rates of 4 mM FA in Hela and Caski cells were 43.7% (Fig. 4a) and 42.2% (Fig. 4b), respectively.

#### The effect of FA on cell autophagy

**Fig. 5:**
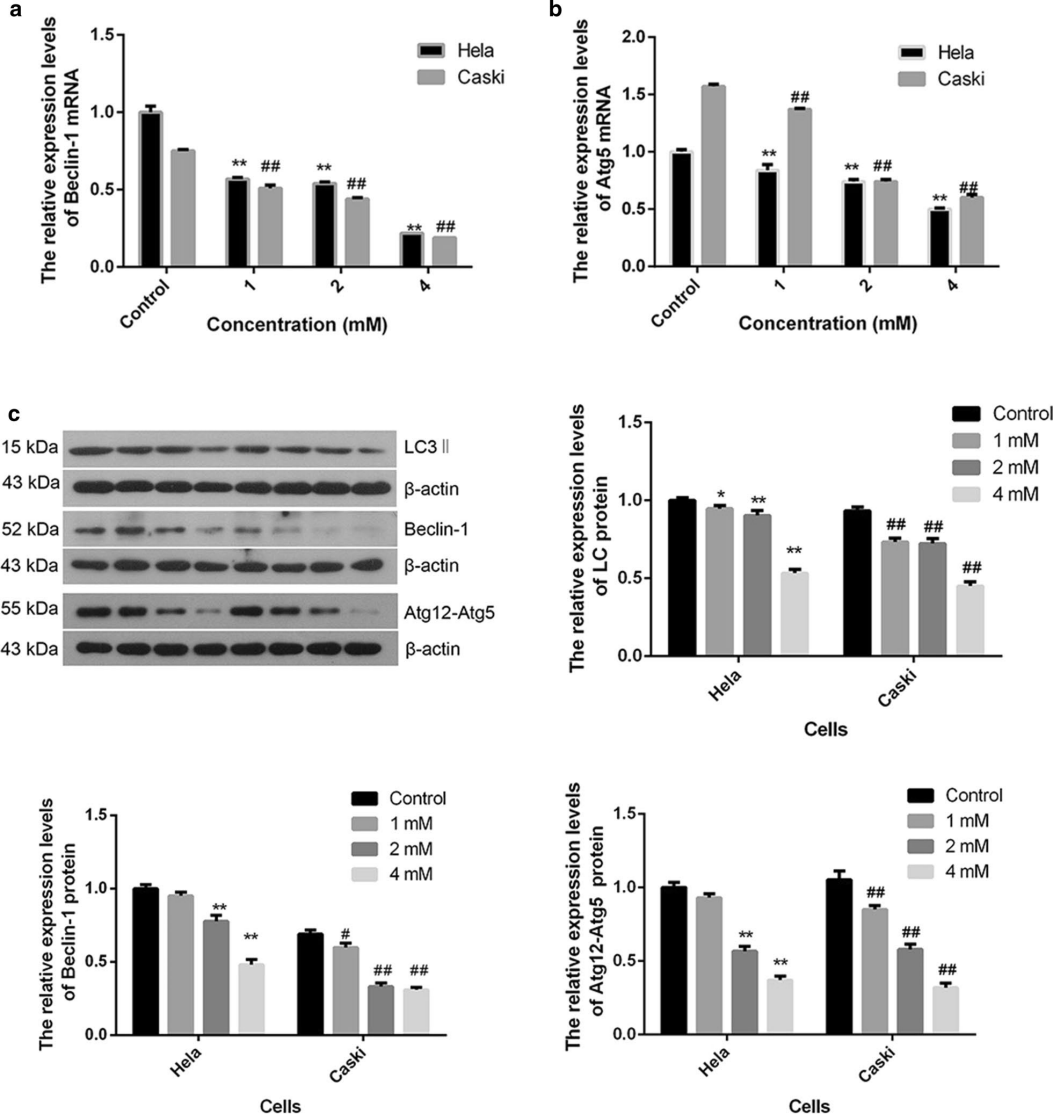
Effect of different concentrations of FA on cell autophagy in Hela and Caski cells. The mRNA expression of Beclin-1, Atg5 in Hela and Caski cells (**a**, **b**); Beclin-1, LC3-II, Atg12-Atg5 protein expression in Hela cells and Caski cells (**c**). Our test is the result of the mark on 15 k, LC3-I and LC3-II are close to a partial overlap, we take LC3-II as the test result. *P < 0.05 indicates that there are significantly different compared with the control group; **P < 0.01

To evaluate the effect of FA on cell autophagy in Hela and Caski cells, the mRNA expressions of autophagy-related genes such as Beclin-1 and Atg5 in the control and FA groups were detected by real-time PCR (Fig. 5). The mRNA expression of Beclin-1 was significantly decreased in both of two cells treatment with 4.0 mM FA when compared with the control group (P < 0.01, Fig. 5a). Similarly, mRNA level of Atg5 was also significantly decreased along with the increasing dosage of FA in Hela and Caski cells (${\text{R}}^{ 2}_{\text{Hela}} = \, 0. 9 6,{\text{ P }} < 0.0 1;{\text{ R}}^{ 2}_{\text{Caski}} = \, 0. 9 3,{\text{ P }} < 0.0 1$, Fig. 5b). Subsequently, the autophagy-related proteins were detected using Western blotting. The relative contents of LC3-II, Beclin-1 and Atg12-Atg5 in Hela cells exposure to 4 mM FA were significantly reduced (P < 0.01, Fig. 5c).

## Discussion

Our study showed that FA had a significant inhibitation effect on Hela and Caski human cervical cancer cells in a concentration-dependent manner. In addition, FA inhibited cell invasion through reducing MMP-9 mRNA expression. FA induced cell apoptosis and G0/G1 phase arrest in Hela and Caski cells through inducing the cell cycle-related proteins expression such as p53 and p21, and reduced Cyclin D1 and Cyclin E levels. Moreover, FA decreased the autophagy-related proteins such as LC3-II, Beclin1 and Atg12-Atg5 in a dose-dependent manner.

Cell cycle is closely related to tumorigenesis. Many tumor-inhibitory factors are involved in cell cycle, such as P53 and its downstream regulators [19]. P21 and P53 genes as the stimulated markers, are involved in cell cycle and apoptosis [20]. P21 is a controller of the G1 and S phases of the cell cycle progression, and thus the overexpression of P21 occurs to repair cell cycle arrest in injured cells [21]. Additionally, P53 protein is a transcription factor that plays an important role in cell growth, DNA repair and cell apoptosis [22]. If P53 gene is downexpressed, the risk of tumorigenesis will increase due to the increased number of impaired DNA [22]. The loss-of-function mutation in the P53 gene contributes to the development of the tumor, and CD44 expression is usually inhibited by the binding of P53 and CD44 promoters. Therefore, an increased expression of CD44 was detected in the mutant P53 tumor cells [23]. One of the cell surface markers associated with cancer stem cells (CSCs) in several types of tumors [24, 25]. Another metabolic heterogeneity leads to the inability to produce the same therapeutic effect on whole cancer cells, and cancer stem cells have shown to cause With several biological properties of conventional anti-tumor therapeutics, metabolic programming is crucial for CSCs to maintain unlimited self-renewal potential and over-adaptation to rapid changes in the tumor microenvironment [26–28], due to the presence of CSCs leading to intratumoral Heterogeneity is the main reason why we cannot induce the same therapeutic effect in whole cancer cells [29]. CSCs are likely to contribute to the formation of minimal residual disease (MRD) [30, 31], and MRD is expected to be at potential recurrence and distant Transfer plays an important role [32]. Similarly, P53 and P21 proteins levels were increased in FA groups. Moreover, downexpression of the cell cycle-related proteins such as cyclinD1 and cyclin E and the inhibition of G1/S can lead to cell cycle arrest [33]. In this study, the levels of cyclin D1 and cyclin E1 proteins were decreased and the levels of P53 and P21 proteins were up-regulated in FA-treated Hela and Caski cells, indicating that FA induced the G1/S cell cycle arrest.

The effect of MMP-9 on tumorigenesis and target therapy is well known [34], which degrades collagen and increases the bioavailability of vascular endothelial growth factor (VEGF) and transforming growth factor-beta (TGF-β) [35]. Activation of MMP-9 led to the cell migration, and the upregulated MMP-9 are associated with invasion, metastasis and poor prognosis in different types of cancers such as colon, ovarian and prostate cancer [36–38]. Metastasis is an important step in the progression of tumors, revealing the metastasis of malignant cells from the original site to distant organs and tissues. Epithelial-mesenchymal transition (EMT) is an important process in cancer cell metastasis and cell invasion, in which epithelial cells enhance resistance to apoptosis, enhance migration and invasiveness [25]. MMP-9 is also closely related to the metastasis of malignant tumors, so we studied the changes of MMP-9 in cervical cancer HeLa cells and Caski cells under the action of FA. In this study, the expression of MMP-9 was decreased in FA groups. Besides, FA inhibited the cell invasion. Therefore, FA inhibited the cell invasion in Hela and Caski cervical cancer cells through reducing the expression of MMP-9.

Autophagy is a double-edged sword for cancer. Studies on drug relocation have shown that “conventional” agents used to treat diseases other than cancer can have antitumor therapeutic effects through activation or suppression of autophagy, and some against autophagy. The latest advances in novel treatment strategies to treat or prevent malignancy [39]. There are studies that have found that ferulic acid has been used in patients with diseases other than malignant tumors. Autophagy can protect cells by inhibiting apoptosis or necrosis, and also promote cell death in coordination with apoptosis. Additionally, autophagy can also induce apoptosis [40]. As a specific marker of autophagosome formation, LC3 exist in the form of LC3-I and LC3-II when autophagy was inactivated or activated [41]. In addition, Beclin-1 is the first mammalian autophagy gene, which may drive the formation of autophagosome by binding to VPS34 [42]. Beclin-1 is an important modifier of autophagy and is closely related to tumorigenesis. Autophagy often involved in biological process such as tumor progression and chemoresistance through constituting a stress adaptation that avoids cell death [43]. It has been reported that LC3-II and Beclin-1 are prognostic factors of various human cancers. The downexpression of Beclin-1 is ovarian epithelial cancer associated with prognosis in ovarian cancer [44], and the expression of LC3-II is associated with a good prognosis of hepatocellular carcinoma [45]. Moreover, tumor suppressor P53 can also be induced autophagy death in cancer cells [46]. Among them, two ubiquitin-like conjugate systems were required during the formation of autophagosome, such as the Atg12 and LC3-II systems. The LC3-II is downstream of the Atg12 system, the Atg12 coupled to Agt5 to form an irreversible Atg12-Atg5 complex [47]. Therefore, this study showed that FA inhibited autophagy through reducing the levels of LC3-II, Beclin-1 and Atg12-Atg5 proteins.

In summary, FA has a significant inhibitory effect on human Hela and Caski cervical cancer cells. FA can also significantly inhibit cell proliferation and invasion. It might be acted as an anti-cancer drug through inhibiting the autophagy and inducing cell cycle arrest in human cervical carcinoma cells. This research provides a theoretical basis for the treatment of human cervical cancer using FA. However, the molecular mechanism is not yet enough comprehensive, and further study is needed.

## Conclusion

In short, we conclude that the anticancer mechanism of ferulic acid is related to the autophagy and cell cycle of Hela and Caski cervical cancer cell lines.

## Abbreviation

FA: ferulic acid (4-hydroxy-3-methoxycinnamic acid)

## References

[1] Ginsburg O, Bray F, Coleman MP, Vanderpuye V, Eniu A, Kotha SR, Sarker M, Huong TT, Allemani C, Dvaladze A (2016). The global burden of women’s cancers: a grand challenge in global health. Lancet.

[2] Ferlay J, Soerjomataram I, Dikshit R, Eser S, Mathers C, Rebelo M, Parkin DM, Forman D, Bray F (2015). Cancer incidence and mortality worldwide: sources, methods and major patterns in GLOBOCAN 2012. Int J Cancer.

[3] Tran NP, Hung CF, Roden R, Wu TC (2014). Control of HPV infection and related cancer through vaccination. Recent Results Cancer Res.

[4] De MC, Plummer M, Vignat J, Franceschi S (2017). Worldwide burden of cancer attributable to HPV by site, country and HPV type. Int J Cancer.

[5] Chen T-C, Huang H-J, Wang T-Y, Yang L-Y, Chen C-H, Cheng Y-M, Liou W-H, Hsu S-T, Wen K-C, Ou Y-C (2015). Primary surgery versus primary radiation therapy for FIGO stages I-II small cell carcinoma of the uterine cervix: a retrospective Taiwanese Gynecologic Oncology Group study. Gynecol Oncol.

[6] Einhorn N, Tropé C, Ridderheim M, Boman K, Sorbe B, Cavallin-Ståhl E (2004). A systematic overview of radiation therapy effects in ovarian cancer. Acta Radiol Ther Phys Biol.

[7] Magge RS, Deangelis LM (2015). The double-edged sword: neurotoxicity of chemotherapy. Blood Rev.

[8] Kumar N, Pruthi V (2014). Potential applications of ferulic acid from natural sources. Biotechnol Rep.

[9] Xi J, Luo S (2016). The mechanism for enhancing extraction of ferulic acid from Radix *Angelica sinensis* by high hydrostatic pressure. Sep Purif Technol.

[10] Dong G-C, Kuan C-Y, Subramaniam S, Zhao J-Y, Sivasubramaniam S, Chang H-Y, Lin F-H (2015). A potent inhibition of oxidative stress induced gene expression in neural cells by sustained ferulic acid release from chitosan based hydrogel. Mater Sci Eng.

[11] Fang L, Chen M, Liu Z, Fang X, Gou S, Chen L (2016). Ferulic acid–carbazole hybrid compounds: combination of cholinesterase inhibition, antioxidant and neuroprotection as multifunctional anti-Alzheimer agents. Bioorg Med Chem.

[12] Zhang X-d WuQ, S-h Yang (2017). Ferulic acid promoting apoptosis in human osteosarcoma cell lines. Pak J Med Sci.

[13] Eroğlu C, Seçme M, Bağcı G, Dodurga Y (2015). Assessment of the anticancer mechanism of ferulic acid via cell cycle and apoptotic pathways in human prostate cancer cell lines. Tumor Biol.

[14] Markiewicz-Żukowska R, Naliwajko SK, Bartosiuk E, Moskwa J, Isidorov V, Soroczyńska J, Borawska MH (2013). Chemical composition and antioxidant activity of beebread, and its influence on the glioblastoma cell line (U87MG). J Apic Sci.

[15] Hirata A, Murakami Y, Atsumi T, Shoji M, Ogiwara T, Shibuya K, Ito S, Yokoe I, Fujisawa S (2005). Ferulic acid dimer inhibits lipopolysaccharide-stimulated cyclooxygenase-2 expression in macrophages. In Vivo.

[16] Prasad NR, Srinivasan M, Pugalendi K, Menon VP (2006). Protective effect of ferulic acid on γ-radiation-induced micronuclei, dicentric aberration and lipid peroxidation in human lymphocytes. Mutat Res/Genet Toxicol Environ Mutagen.

[17] Karthikeyan S, Kanimozhi G, Prasad NR, Mahalakshmi R (2011). Radiosensitizing effect of ferulic acid on human cervical carcinoma cells in vitro. Toxicol In Vitro.

[18] Fahrioğlu U, Dodurga Y, Elmas L, Seçme M (2016). Ferulic acid decreases cell viability and colony formation while inhibiting migration of MIA PaCa-2 human pancreatic cancer cells in vitro. Gene.

[19] Evan GI, Vousden KH (2001). Proliferation, cell cycle and apoptosis in cancer. Nature.

[20] Chen QM, Liu J, Merrett JB (2000). Apoptosis or senescence-like growth arrest: influence of cell-cycle position, p53, p21 and bax in H2O2 response of normal human fibroblasts. Biochem J.

[21] Mikule K, Delaval B, Kaldis P, Jurcyzk A, Hergert P, Doxsey S (2007). Loss of centrosome integrity induces p38—p53—p21-dependent G1—S arrest. Nat Cell Biol.

[22] Schärer E, lggo R (1992). Mammalian p53 can function as a transcription factor in yeast. Nucleic Acids Res.

[23] Godar S (2008). Growth-inhibitory and tumor-suppressive functions of p53 depend on its repression of CD44 expression. Cell.

[24] Yoshida GJ, Fuchimoto Y, Osumi T (2012). Li-Fraumeni syndrome with simultaneous osteosarcoma and liver cancer: Increased expression of a CD44 variant isoform after chemotherapy. BMC Cancer.

[25] Yoshida GJ, Saya H (2013). Inversed relationship between CD44 variant and c-Myc due to oxidative stress-induced canonical Wnt activation. Biochem Biophys Res Commun.

[26] Mihaylova MM, Shaw RJ (2011). The AMPK signalling pathway coordinates cell growth, autophagy and metabolism. Nat Cell Biol.

[27] Metcalf DJ, Garcia-Arencibia M, Hochfeld WE, Rubinsztein DC (2012). Autophagy and misfolded proteins in neurodegeneration. Exp Neurol.

[28] Jimenez-Sanchez M, Thomson F, Zavodszky E, Rubinsztein DC (2012). Autophagy and polyglutamine diseases. Prog Neurobiol.

[29] Wileman T (2013). Autophagy as a defence against intracellular pathogens. Essays Biochem.

[30] Meads MB, Gatenby RA, Dalton WS (2009). Environment-mediated drug resisitance:a major contributor to minimal residual disease. Nat Rev Cancer.

[31] Yoshida Go J (2017). The heterogeneity of cancer stem-like cells at the invasive front. Cancer Cell Int.

[32] Yoshida Go J (2015). Metabolic reprogramming:the emerging concept and associated therapeutic strategies. J Exp Clin Cancer Res.

[33] Prall OW, Sarcevic B, Musgrove EA, Watts CK, Sutherland RL (1997). Estrogen-induced activation of Cdk4 and Cdk2 during G1-S phase progression is accompanied by increased cyclin D1 expression and decreased cyclin-dependent kinase inhibitor association with cyclin E-Cdk2. J Biol Chem.

[34] Bauvois B (2012). New facets of matrix metalloproteinases MMP-2 and MMP-9 as cell surface transducers: outside-in signaling and relationship to tumor progression. Biochim Biophys Acta Rev Cancer.

[35] Behzadian MA, Wang X-L, Windsor LJ, Ghaly N, Caldwell RB (2001). TGF-β increases retinal endothelial cell permeability by increasing MMP-9: possible role of glial cells in endothelial barrier function. Invest Ophthalmol Vis Sci.

[36] Legrand C, Polette M, Tournier J-M, de Bentzmann S, Huet E, Monteau M, Birembaut P (2001). uPA/plasmin system-mediated MMP-9 activation is implicated in bronchial epithelial cell migration. Exp Cell Res.

[37] Salem N, Kamal I, Al-Maghrabi J, Abuzenadah A, Peer-Zada AA, Qari Y, Al-Ahwal M, Al-Qahtani M, Buhmeida A (2016). High expression of matrix metalloproteinases: mMP-2 and MMP-9 predicts poor survival outcome in colorectal carcinoma. Future Oncol.

[38] Yao Z, Yuan T, Wang H, Yao S, Zhao Y, Liu Y, Jin S, Chu J, Xu Y, Zhou W (2017). MMP-2 together with MMP-9 overexpression correlated with lymph node metastasis and poor prognosis in early gastric carcinoma. Tumor Biol.

[39] Yoshida GJ (2017). Therapeutic strategies of drug repositioning targeting autophagy to induce cancer cell death:from pathophysiology to treatment. J Hematol Oncol..

[40] Maiuri MC, Zalckvar E, Kimchi A, Kroemer G (2007). Self-eating and self-killing: crosstalk between autophagy and apoptosis. Nat Rev Mol Cell Biol.

[41] Tanida I, Ueno T, Kominami E (2008). LC3 and Autophagy. Methods Mol Biol.

[42] Funderburk SF, Wang QJ, Yue Z (2010). The Beclin 1-VPS34 complex–at the crossroads of autophagy and beyond. Trends Cell Biol.

[43] Wei Y, Zou Z, Becker N, Anderson M, Sumpter R, Xiao G, Kinch L, Koduru P, Christudass C, Veltri R (2013). EGFR-Mediated Beclin 1 phosphorylation in autophagy suppression, tumor progression, and tumor chemoresistance. Cell.

[44] Cai M, Hu Z, Liu J, Gao J, Liu C, Liu D, Tan M, Zhang D, Lin B (2014). Beclin 1 expression in ovarian tissues and its effects on ovarian cancer prognosis. Int J Mol Sci.

[45] Lee YJ, Hah YJ, Na KY, Kang KJ, Hwang JS, Chung WJ, Cho KB, Park KS, Kim ES, Seo H (2013). Correction: the autophagy-related marker lc3 can predict prognosis in human hepatocellular carcinoma. PLoS ONE.

[46] Maiuri MC, Galluzzi L, Morselli E, Kepp O, Malik SA, Kroemer G (2010). Autophagy regulation by p53. Curr Opin Cell Biol.

[47] Otomo C, Metlagel Z, Takaesu G, Otomo T (2013). Structure of the human ATG12 ~ ATG5 conjugate required for LC3 lipidation in autophagy. Nat Struct Mol Biol.

